# Over-Expressing Mitofusin-2 in Healthy Mature Mammalian Skeletal Muscle Does Not Alter Mitochondrial Bioenergetics

**DOI:** 10.1371/journal.pone.0055660

**Published:** 2013-01-31

**Authors:** James S. V. Lally, Eric A. F. Herbst, Sarthak Matravadia, Amy C. Maher, Christopher G. R. Perry, Renée Ventura-Clapier, Graham P. Holloway

**Affiliations:** 1 Department of Human Health and Nutritional Sciences, University of Guelph, Guelph, Ontario, Canada; 2 INSERM U769, Université Paris-Sud, Châtenay-Malabry, Paris, France; Inserm, France

## Abstract

The role of mitofusin-2 (MFN-2) in regulating mitochondrial dynamics has been well-characterized in lower order eukaryotic cell lines through the complete ablation of MFN-2 protein. However, to support the contractile function of mature skeletal muscle, the subcellular architecture and constituent proteins of this tissue differ substantially from simpler cellular organisms. Such differences may also impact the role of MFN-2 in mature mammalian muscle, and it is unclear if minor fluctuations in MFN-2, as observed in response to physiological perturbations, has a functional consequence. Therefore, we have transiently transfected MFN-2 cDNA into rat tibialis anterior muscle to determine the effect of physiolgically relevant increases in MFN-2 protein on mitochondrial bioenergetics. Permeabilized muscle fibres generated from muscle following MFN-2-transfection were used for functional assessments of mitochondrial bioenergetics. In addition, we have further established a novel method for selecting fibre bundles that are positively transfected, and using this approach transient transfection increased MFN-2 protein ∼2.3 fold in selected muscle fibres. However, this did not alter maximal rates of oxygen consumption or the sensitivity for ADP-stimulated respiration. In addition, MFN-2 over-expression did not alter rates of H_2_O_2_ emission. Altogether, and contrary to evidence from lower order cell lines, our results indicate that over-expressing MFN-2 in healthy muscle does not influence mitochondrial bioenergetics in mature mammalian skeletal muscle.

## Introduction

Over the past decade the physiological role of mitochondria has been expanded. They are most well known for providing ATP for cellular processes, however it now appears that mitochondria are dynamic organelles, both with respect to their structure and function [1], [2]. Within muscle cells mitochondria exist as both discrete organelles and as elongated tubules [3], [4], and the mitochondrial reticulum network appears to continually undergo fusion and fission reactions to influence the overall morphology of mitochondria, a process that is thought to effect function of this organelle. Several proteins have been implicated in these events, including mitofusin-1 (MFN-1), MFN-2 and autosomal dominant optical atrophy-1 (OPA1) which coordinate fusion, whereas dynamim-related protein 1 (DRP1) and fission-1 (Fis-1) appear to regulate fission [5], [6], [7], [8], [9].

In yeast, mitochondrial fusion requires a mitochondrial transmembrane GTPase encoded by the *fzo* gene, as repression of the *fzo* gene creates fragmented mitochondria [10], [11], [12]. Other yeast proteins, such as Dnm1p and Mgm1p, regulate mitochondrial fission [13], [14], [15]. The role of MFN-2 in regulating mitochondrial dynamics has been well characterized in various cell lines, as repression of MFN-2 in L6E9 myotubes and 10T1/2 cells results in fragmented and disorganized mitochondria. Conversely, over-expression of MFN-2 in HeLa cells, L6E9 myotubes and COS cells results in perinuclear clustering of mitochondria [16], [17], [18]. In addition, within various cell lines, including L6E9 myotubes and HeLa cells, decreasing the expression of MFN-2 effects mitochondrial biochemical processes, including reducing the oxidation of various substrates and rates of oxygen consumption [7], [9], [16], [17]. Work in lower eukaryotic cells has also shown that mitochondria can migrate within these models [19], and the subsequent fusion events allows for the sharing of matrix constituents and signal transduction between organelles [20], which may be advantageous for mitochondrial bioenergetics [19]. While mammalian cells contain homologous proteins, suggesting the regulation of the mitochondrial reticular morphology is evolutionarily conserved, the functional role of these proteins in mature mammalian skeletal muscle is not yet known. This tissue differs markedly from lower eukaryotic cells with respect to its architecture, which supports its physiologic contractile function. The skeletal muscle contractile apparatus, which is virtually absent in cell lines, but comprises much of the myocellular volume *in vivo*, possibly limits mitochondrial migration. Instead communication among subsarcolemmal and intermyofibrillar mitochondria may occur via tubular filaments that penetrate the muscle fibres [7], [21]. These tubular filaments may optimize conductance of membrane potential from subsarcolemmal mitochondria, where oxygen and oxidizable substrates are most easily available, to intermyofibrillar mitochondrial regions where the delivery of these compounds may be compromised, but where ATP requirement is substantial during muscle contraction [7], [21].

In view of the structural and organizational differences between mature mammalian skeletal muscle and various cell lines, it is uncertain whether the functional roles of selected mitochondrial dynamic proteins identified in lower order eukaryotic cells can be extrapolated to mature mammalian skeletal muscle. In support of this, MFN-1^−/−^MFN-2^+/−^ mice do not show reductions in mitochondrial content or severe alterations in mitochondrial morphology [22]. In addition, contrary to work in various cell lines [7], [9], [17], MFN-1 ablation in mice does not reduce oxygen consumption or rates of ATP production in muscle [22] and ablation of MFN-2 in the heart does not effect proton motive force or overal performance of the heart, suggesting MFN-2 deficiency does not impact ATP production or mitochondrial bioenergetics [23]. Combined, these data suggest that research pertaining to the functional role of MFN-2 conducted in selected cell lines cannot be extrapolated to mature mammalian muscle, a notion further recognized in other tissues, as ablating MFN-2 in neonatal cardiac myocytes results in smaller mitochondria, while the same approach in adult cardiac myocytes results in larger mitochondria [24].

Clearly, the identification of the functional role of MFN-2 in regulating mammalian skeletal muscle mitochondrial bioenergetics has not been fully delineated. Given that conditional ablation of MFN-1 does not dramatically affect mitochondrial morphology or respiration [22], we have taken an alternative approach to examine the role of MFN-2 in mature mammalian skeletal muscle. Specifically, we have transiently transfected MFN-2 cDNA into rodent muscle to determine the effect on mitochondrial bioenergetics in permeabilized muscle fibres. Our results do not support the widely-held belief, based on studies using cell lines, that over-expressing MFN-2 influences mitochondrial bioenergetics in healthy mammalian skeletal muscle.

## Materials and Methods

### Animals

Female Sprague-Dawley rats bred on site at the University of Guelph were used at 10 weeks of age. All animals were housed in a climate and temperature controlled room, on a 12∶12 hour light-dark cycle, with rat chow and water provided *ad libitum* prior to the start of experiments. In all experiments animals were anaesthetized with an intraperitoneal injection of sodium pentobarbital (60 mg kg^−1^) before muscle tissue was harvested. All facets of this study were approved by the University of Guelph Animal Care Committee, and conform to the guide for the care and use of laboratory animals published by the US National Institutes of Health.

### Transient electrotransfection of MFN-2

Electrotransfection experiments were performed as previously described by us [25], [26] and others [27], [28]. The rat MFN-2 cDNA clone in pCMVsport6 expression vector was purchased from Invitrogen (Burlington, ON, Canada). Thereafter, in brief, animals were anesthetized with isoflurane, and the tibialis anterior muscle was electrotransfected with i) pCMVsport6 plasmid containing MFN-2 cDNA (250 µg in 0.45% saline), or ii) empty plasmid (250 µg cDNA in 0.45% saline). Rats were provided with an analgesic (Temgesic) and allowed to recover for either 1 or 2 weeks (n = 3 for both time points). The red portion of the tibialis anterior muscle was utilized for all experiments as 1) this muscle has a high oxidative capacity (∼75% type I and IIa fibres [29]), and 2) the superficial anatomical position of this muscle negates the need for invasive surgery, decreasing the risk of infection and a strong immune response. Data were not different between time points and therefore the data have been combined to increase statistical power.

### Isolated mitochondrial bioenergetics from skeletal muscle

Differential centrifugation was used to obtain mitochondria as previously published [30]. In brief, muscle was minced with scissors, homogenized with a motor driven teflon pestel, and centrifuged at 800 g for 10 minutes. The supernatant was subsequently centrifuged twice at 10,000 g for 10 minutes, and the pellet resuspended for bioenergetic assessments. Rates of oxygen consumption were measured in isolated mitochondria (0.2 mg/ml) at 25°C by high-resolution respirometry (Oroboros Oxygraph-2 k, Innsbruck, Austria) in standard incubation medium (120 mM KCl, 1 mM EGTA, 5 mM KH_2_PO_4_, 5 mM MgCl_2_ and 5 mM HEPES; pH 7.4). The sequential respiration protocol consisted of determining state IV (leak respiration) respiration in the presence of 5 mM pyruvate+2 mM malate, 100 µM ADP to determine P/O ratios, 5 mM ADP to determine maximal state III (maximum phosphorylating) respiration, 10 mM glutamate to determine maximal complex I supported respiration, and 10 mM succinate to determine maximal coupled oxygen consumption. Mitochondrial hydrogen peroxide (H_2_O_2_) emission was determined fluorometrically (Lumina, Thermo Scientific) in a constantly stirring cuvette at 25°C (peltier controlled), in 300 µl of a standard reaction media (in mM; 100K-MES, 35 KCl, 1 EGTA, 5 K_2_HPO_4_ and 3 MgCl_2_6H_2_O, 5 mg/ml BSA, 0.05 pyruvate and 0.02 malate (pH 7.4)) supplemented with 10 µM Amplex red, 0.5 U/ml horseradish peroxidase, 10 mM succinate, 10 µg/ml oligomycin and 40 U/ml Cu,Zn-SOD [31]. The rate of H_2_O_2_ emission was calculated from the slope (F/min), after subtracting the background, from a standard curve established with the same reaction conditions and normalized to mitochondrial protein (nmol/min/mg mitochondrial protein).

### Transient electrotransfection of lac-Z

The β-galactosidase expression vector (pSV-beta-galactosidase) was purchased from promega (Madison, WI, USA). The tibialis anterior muscle of animals were then transfected with 250 µg cDNA (n = 3) as described above. Thereafter, permeabilized muscle fibres were isolated from the red portion of the muscle (as described below), and incubated in the presence of 1 mg/ml X-gal at room temperature for 2 hours. Muscle fibre bundles were then imaged using an Infinity-2 camera (Electron Microscopy Sciences, Hatfield, PA, USA) mounted on a stereomicroscope (Zeiss Stemi 2000, Oberkochen, Germany).

### Mitochondrial bioenergetics in permeabilized fibres

The preparation of saponin permeabilized fibres and bioenergetic assessments were adopted from previous publications [31], [32], [33], [34], as we have reported [35]. Fibre bundles (∼2 mg) were separated with fine forceps under a binocular dissecting microscope in BIOPS buffer containing, CaK_2_EGTA (2.77 mM), K_2_EGTA (7.23 mM), Na_2_ATP (5.77 mM), MgCl_2_*6H_2_O (6.56 mM), Na_2_Phosphocreatine (15 mM), Imidazole (20 mM), Dithiothreitol (0.5 mM) and MES (50 mM). Following separation, fibre bundles were placed in BIOPS containing 40 µg/ml saponin, agitated for 30 min and then washed in respiration buffer (MIRO5) containing EGTA (0.5 mM), MgCl_2_*6H_2_O (3 mM), K-lactobionate (60 mM), KH_2_P0_4_ (10 mM), HEPES (20 mM), Sucrose (110 mM) and fatty acid free BSA (1 g/L). Fibres were left in cold MIRO5 until respiration analysis. Mitochondrial respiration was measured in permeabilized fibers by high-resolution respirometry (Oroboros Oxygraph-2 k, Innsbruck, Austria) at 37°C and room air saturation in the presence of 25 µM blebbistatin [33]. The respiration protocol consisted of state IV respiration in the presence of 5 mM pyruvate+2 mM malate, the subsequent titration of ADP to maximal state III respiration (4 mM), and then the sequential addition of 10 mM glutamate and 10 mM succinate. Maximal uncoupled respiration was determined by titrating FCCP. Rates of mitochondrial H_2_O_2_ emission were determined at 37°C in an identical fashion to the methods outlined for isolated mitochondria with the addition of 25 µM blebbistatin. After functional assessments, permeabilized fibres were recovered, dried and weighed. Thereafter, fibres were used for Western blotting to determine which fibres over-expressed MFN-2, as described below.

### Digestion of permeabilized fibres for Western blotting

The procedure for extracting protein from dried permeablized fibre bundles was adapted from methods previously published from Western blotting on single fibres [36]. Proteins were extracted using solubilizating buffer (0.125 M Tris-Cl, pH 6.8, 4% SDS, 10% glycerol, 4 M urea, 10% mercaptoethanol, and 0.001% bromophenol blue). Pilot experiments determined that the protein extraction was linear at all resuspension volumes tested (0.5 µl–1.5 µl per 5 mg fibre dry weight) as determined by Western blotting for MFN-2, cytochrome c oxidase complex IV (COXIV) and CD36 (r>0.95 for all three proteins). Based on these experiments, dried muscle fibres were resuspended in 1 µl of solubilization buffer per 5 µg dry permeabilized fibre weight, freeze thawed once, and allowed to incubated at room temperature overnight while shaking. After overnight incubation 1.5 volumes of 50 mM Tris-Cl pH 6.8 was added to the sample and 20 µl of the sample loaded for analysis by SDS-PAGE as described below.

### Western blotting

Samples were separated by electrophoresis on SDS-polyacrylamide gels and transferred to polyvinylidene difluoride membranes as we have previously reported [37]. Commercially available antibodies were used to detect MFN-2 (Abnova, Hornby, On, Canada) and COXIV (Invitrogen). All samples for a given protein were transferred and developed on the same membrane to limit variation. Blots were quantified using chemiluminescence and the FluorChem HD imaging system (Alpha Innotech, Santa Clara, CA, USA).

### Statistics

The Km for ADP was determined through the Michaelis-Menten enzyme kinetics - fitting model (Y = Vmax*X/(Km+X)), where X = [free ADP; ADP_f_] and Y = *J*O_2_ at [ADP_f_], using Prism (GraphPad Software, Inc., La Jolla, CA, USA), as published previously [33]. All other parameters were analyzed using a Mann Whitney U test. Data are presented as means +/− S.E.M., and significance was accepted at *P*<0.05.

## Results and Discussion

**Figure 1:**
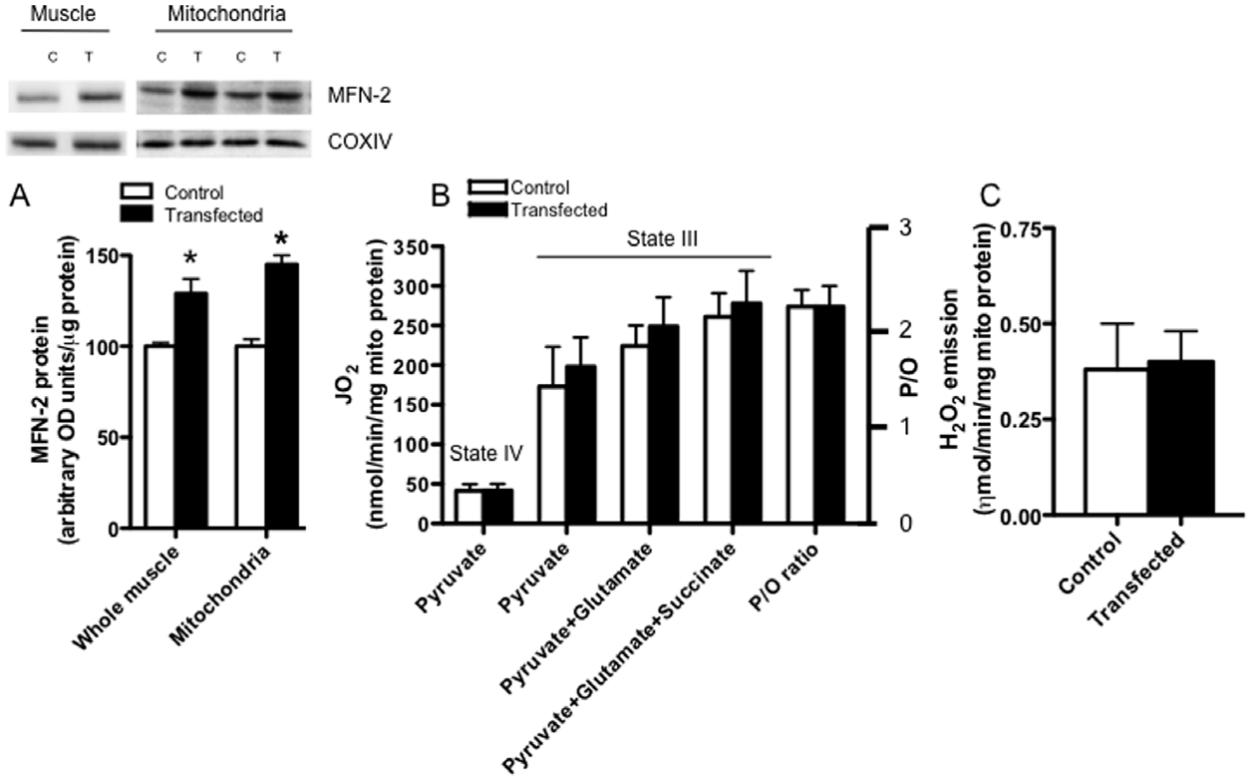
The effect of increasing MFN-2 protein bioenergetics in isolated mitochondria. Transient transfection increased mitofusin-2 (MFN-2) protein in muscle homogenates and in isolated mitochondria (A), but this did not alter state IV (without ADP) or state III (with ADP) respiration rates in the presence of various substrates (B). In addition, the ratio of ADP consumed per oxygen atom (P/O ratio) was not altered (B), nor were rates of hydrogen peroxide (H_2_O_2_) emission (C), following MFN-2 transfection. Results represent means ± S.E.M.; n = 4; * P<0.05 compared to control.

In the current study we transiently over-expressed MFN-2 protein to study the effects on mitochondrial bioenergetics in rat muscle. Before transfection experiments commenced, the cDNA sequence was verified to be correct. It was also important to first show that transient transfection of MFN-2 cDNA increased MFN-2 protein content at the whole muscle level (+29%; P<0.05; Fig. 1A) and in isolated mitochondria (+40%; P<0.05; Fig. 1A), providing evidence that MFN-2 was appropriately over-expressed within this organelle.

### Bioenergetic effects of MFN-2 overexpression in isolated mitochondria

To determine the functional consequence of over-expressing MFN-2 we first examined the bioenergetics in isolated mitochondria. Transient transfection of MFN-2 did not alter pyruvate+malate supported state IV or state III respiration rates (Fig. 1B), and therefore the respiratory control ratios were not different (RCR ∼5 for control and transfection; data not shown). Maximal complex I supported respiration (pyruvate+glutamate+malate) and maximal oxidative phosphorylation capacity (pyruvate+glutamate+succinate+malate) were also not altered following MFN-2 over-expression (Fig. 1B). In addition to unaltered maximal respiration rates, the efficiency of mitochondria remained unaltered as evident by constant P/O ratios (Fig. 1B).

To further assess mitochondrial bioenergetics following MFN-2 over-expression, we determined maximal succinate induced H_2_O_2_ emission. As with the other parameters examined, succinate induced H_2_O_2_ emission was not altered by MFN-2 overexpression (Fig. 1C).

### Bioenergetic effects of MFN-2 overexpression in permeabilized muscle fibres

#### Methodological considerations

Given that MFN-2 is thought to influence mitochondrial fusion and therefore its reticulum structure [7], [17], [18], [38], the determination of bioenergetics following MFN-2 transfection in isolated mitochondria may not be appropriate, as the isolation of mitochondria has been shown to alter its reticular architecture [39]. Therefore, we determined mitochondrial bioenergetics in permeabilized muscle fibres, a methodology that retains mitochondrial reticulum structure [39]. However, bioenergetic analyses of permeabilized muscle fibres only require ∼2 mg (wet weight) of muscle. This raises the possibility that muscle fibers will be selected that have not been transfected with MFN-2, as it is well known that in mature mammalian muscle not all muscle fibres are necessarily transfected [27]. Therefore we first needed to establish a methodology that could identify muscle fibres that over-expressed MFN-2, so we could select these fibers for bioenergetic analyses.

### Establishing methodology to select positively transfected muscle fibres

**Figure 2:**
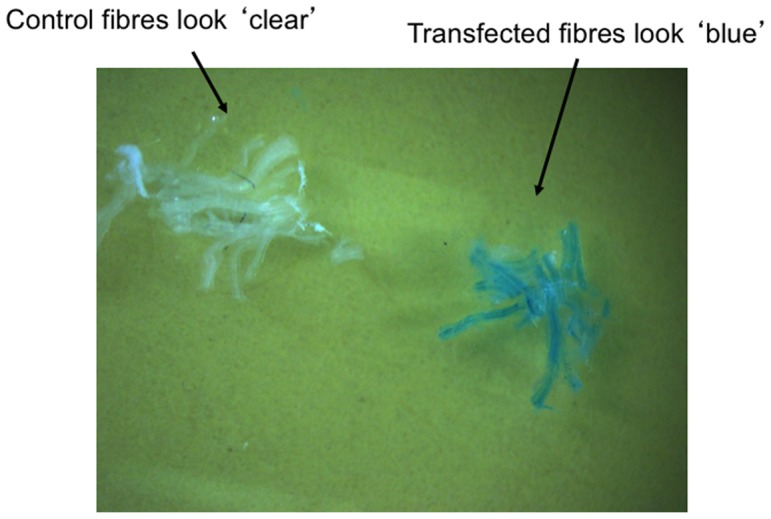
Imaging fibre bundles positively transfected with a vector containing the Lac-Z gene. Permeabilized muscle fibres were generated and exposed to X-gal and then imaged. Fibres positively transfected turned blue, while non-transfected fibres remained clear. Representative images shown from three independent experiments.

As a first approach to select fibres with positive MFN-2 over-expression we aimed to transfect β-galactosidase (lacZ gene) and MFN-2 in a dual-expression vector. However, before subcloning these genes into the dual-expression vector, as proof of principle of the experimental design we transiently transfected the lacZ gene independently into the tibialis anterior muscle and determined 1) if we could detect positively transfected fibres, and 2) if mitochondrial bioenergetics remained stable following this experimental approach. Therefore, two weeks after transfection of the lacZ gene we dissected and permeabilized muscle fibre bundles from various regions of the tibialis anterior muscle and incubated these in the presence of X-gal. Regions containing the lacZ gene turned blue, and therefore were positively transfected (Fig. 2), while non-transfected fibres were not stained and remained white in appearance (Fig. 2), suggesting we could determine which fibres were transfected. The transfected ‘blue’ fibres were then used to determine mitochondrial respiration rates. However, the addition of exogenous ADP did not stimulate respiration in these ‘blue’ fibres, and as a result the respiratory control ratios were very low (1.2±0.17 arbitrary units). This likely occurred as a result of the X-gal product 5,5′-dibromo-4,4′-dichloro-indo, which is insoluble, as trasfection of the lacZ gene without the X-gal substrate did not affect respiration. Therefore, although this approach allowed for the clear and simple identification of transfected fibre bundles, it was not optimal for determining mitochondrial bioenergetics following MFN-2 transfection, and therefore we abandoned utilization of the lacZ gene as a tool to identify transfected fibres.

**Figure 3:**
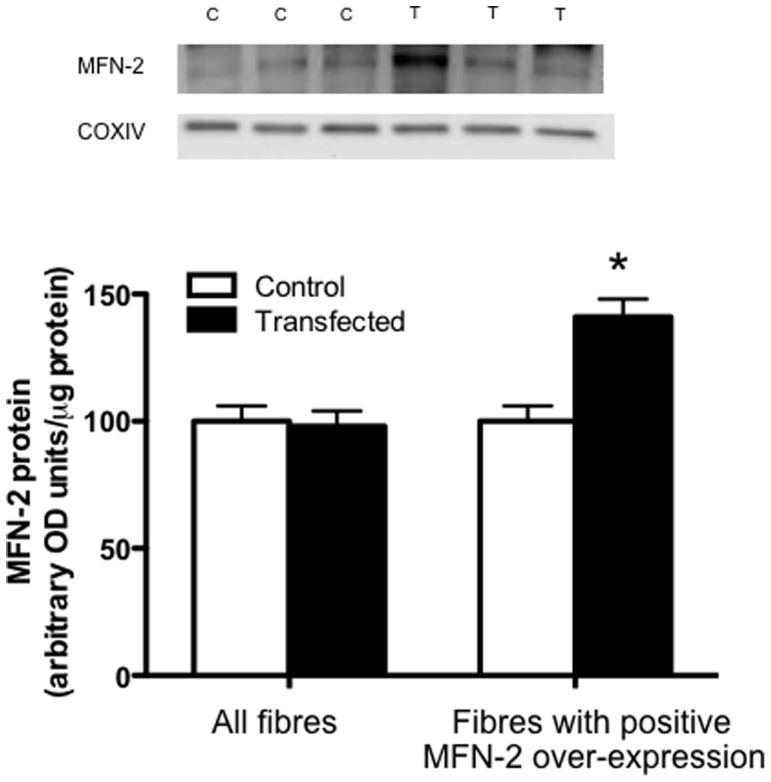
Digestion of fibre bundles following respiration experiments to select fibres positively transfected with MFN-2. A total of 36 fibre bundles were analyzed following MFN-2 transient transfection (T), and 11 of these displayed higher MFN-2 protein relative to all fibres from the contralateral control muscle (C). Results represent means ± S.E.M.; n = 6; * P<0.05 compared to control.

Given the negative effects of utilizing the lacZ gene to identify a region positively transfected an alternative approach was established to confirm the positive over-expression of MFN-2 in fibre bundles. Specfically, we independently transfected MFN-2 cDNA and modified a technique to digest fibres in an SDS buffer [36], such that Western blotting could be performed on permeabilized muscle fibres following functional analysis to determine MFN-2 protein. Using this approach we identified 31% of fibre bundles as positively transfected, which is in line with the expected values given the known transfection efficiency of mature mammalian skeletal muscle [27]. When all fibres were included, MFN-2 protein was increased ∼25% (P<0.05; Fig. 3) following transfection. However, within the transfected muscle eleven fibre bundles expressed MFN-2 to a greater extent compared to all fibre bundles isolated from contralatearal control muscle. In these fibres there was a ∼2.3 fold increase in MFN-2 protein (P<0.05; Fig. 3).

### Respiration in MFN-2 transfected muscle fibres

**Figure 4:**
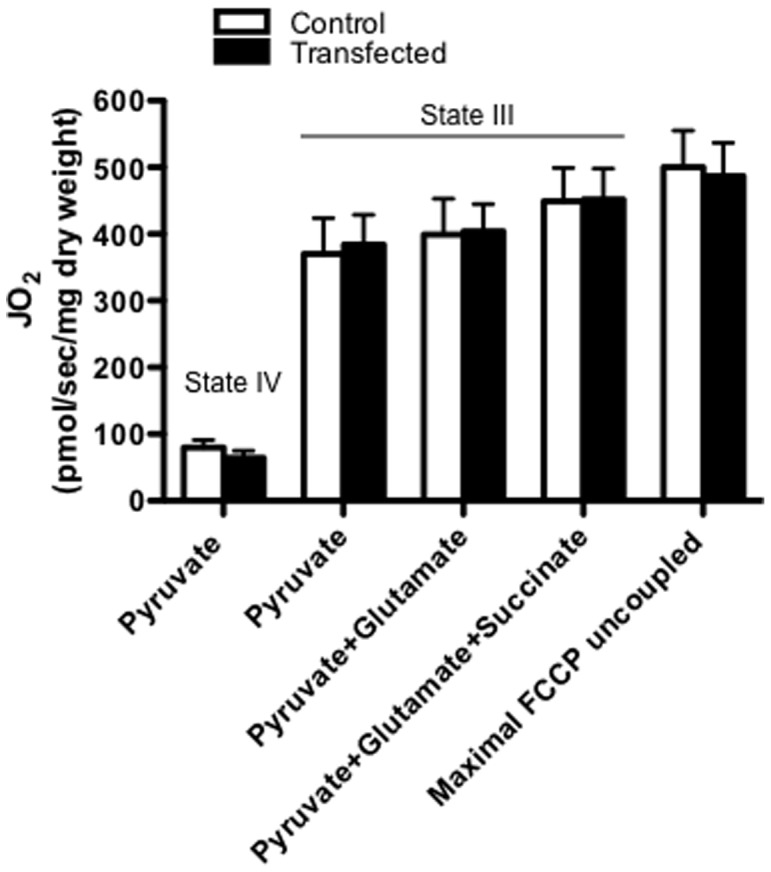
Respiration in permeabilized fibre bundles selected with MFN-2 over-expression. The respiration protocol consisted of state IV respiration in the presence of 5 mM pyruvate+2 mM malate, the subsequent titration of ADP to maximal state III respiration (4 mM), and then the sequential addition of 10 mM glutamate and 10 mM succinate. Maximal uncoupled respiration was determined by titrating FCCP. Results represent means ± S.E.M.; n = 6 independent experiments.

We next determined the effect of increasing MFN-2 protein on the respiration rates in these highly specific MFN-2 transfected muscle fibres (Fig. 4). Transient transfection of MFN-2 in these selected fibres did not alter pyruvate+malate supported state IV or state III respiration rates (Fig. 4), and therefore the respiratory control ratios were not different (control; 4.9±0.4 vs transfection; 5.1±0.7). Maximal complex 1 supported respiration (pyruvate+glutamate+malate), maximal oxidative phosphorylation capacity (pyruvate+glutamate+succinate+malate) and maximal uncoupled respiration (FCCP) were also not altered following MFN-2 over-expression (Fig. 4) suggesting increasing MFN-2 protein does not alter maximal respiration rates in healthy mature mammalian muscle. In L6E9 myotubes, repression of MFN-2 has been shown to alter mitochondrial morphology and the expression of OXPHOS genes [16]. In the current study, despite a greater than 2 fold increase in MFN-2 protein maximal ADP and uncoupled respiration were not different following transient transfection. These data strongly suggest OXPHOS capacity was not altered following transient transfection, as maximal respiration rates are influenced by the expression of OXPHOS genes. The current data clearly show that over-expressing MFN-2 does not alter mitochondrial respiration rates in rat skeletal muscle.

**Figure 5:**
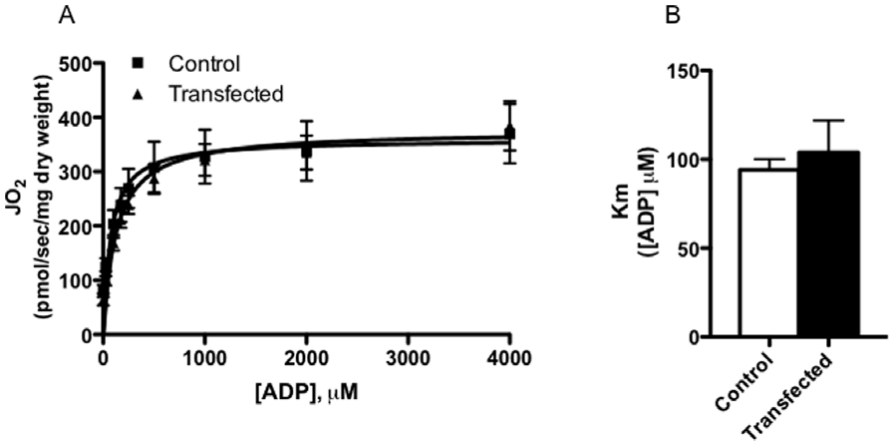
Mitochondrial respiratory sensitivity to ADP in permeabilized fibre bundles selected with MFN-2 over-expression. ADP kinetic analysis was performed in fibre bundles selected for MFN-2 over-expression in the presence of 10 mM pyruvate+5 mM malate and 24 mM creatine. Results represent means ± S.E.M.; n = 6 independent experiments.

The role of the tubular network in mammalian muscle remains unknown, but it has been speculated that mitochondria that penetrate into the deep regions of muscle fibres enable the efficient transfer of the electrical chemical gradient [21], improving the dynamic response of mitochondria to various energetic states. In this respect, maximal state III respiration may not be sensitive enough to detect alterations following MFN-2 over-expression. Therefore, we also determined mitochondrial respiration kinetics in the presence of various ADP concentrations (Fig. 5A), as in the heart decreasing OPA1 content has been shown to affect mitochondrial ADP respiratory kinetics [40]. Respiration was not different in transfected fibres at any ADP concentration studied (25–4000 µM). Hence, the Km for ADP was not different following MFN-2 over-expression (Fig. 5A,B). Therefore, while MFN-2 may be required for normal mitochondrial bioenergetics [7], [16], the current data suggests that increasing MFN-2 protein in healthy muscle does not alter mitochondrial respiration rates.

### Mitochondrial H_2_O_2_ emission in MFN-2 transfected muscle fibres

**Figure 6:**
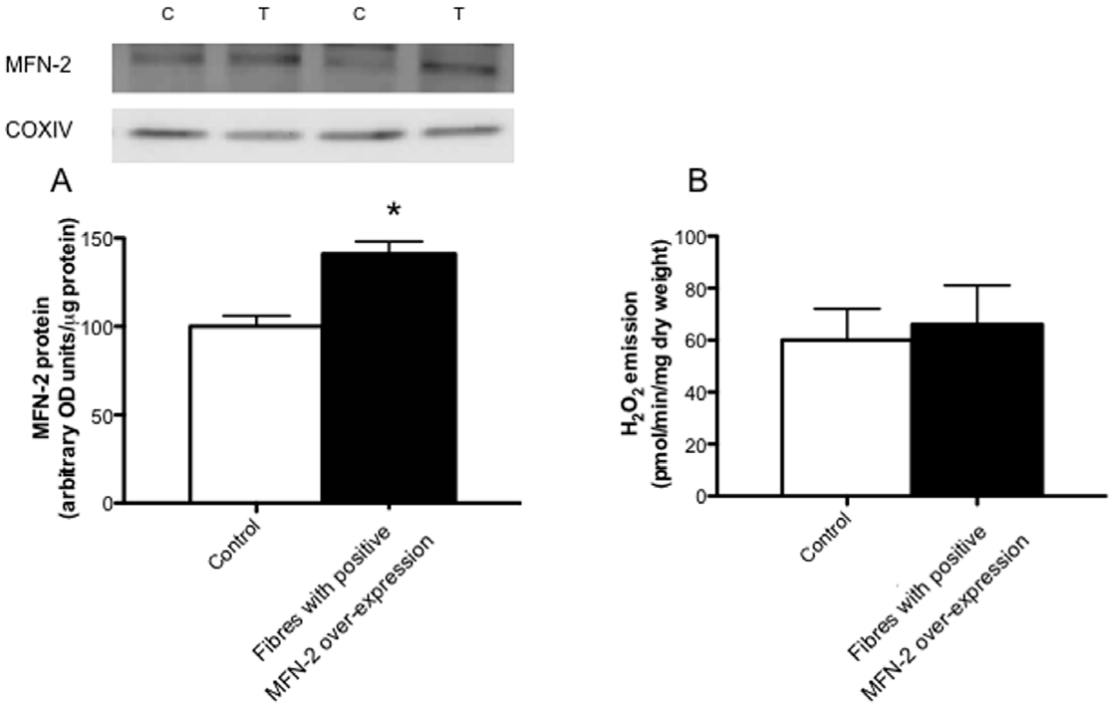
Mitochondrial hydrogen peroxide (H_2_O_2_) emission (B) in permeabilized fibre bundles selected with MFN-2 over-expression (A). A total of 18 fibre bundels were analyzed following MFN-2 transient transfection (T), and 4 of these displayed higher MFN-2 protein relative to all fibres from the contralateral control muscle (C). Mitochondrial hydrogen peroxide (H_2_O_2_) emission was determined fluorometrically (Lumina, Thermo Scientific) in a constantly stirring cuvette at 37°C in a standard reaction media supplemented with 5 µM Amplex red, 0.5 U/ml horseradish peroxidase, 10 mM succinate, 10 µg/ml oligomyocin and 40 U/ml Cu,Zn-SOD and 25 µM blebbistatin. Results represent means ± S.E.M.; n = 6 independent experiments.

Previous work in L6E9 myotubes has shown that knocking-down MFN-2 results in a decrease in membrane potential [7], [16], however ablating MFN-2 increased oxidative stress in the liver of mice [41]. Therefore to further assess the influence of over-expressing MFN-2 on mitochondrial bioenergetics we determined rates of mitochondrial succinate induced H_2_O_2_ emission, a physiologically relevant parameter that is directly regulated by membrane potential. For these experiments a total of 18 fibre bundles were isolated from the same 6 MFN-2 transfected/control muscles used in the respiration experiments. Among these, 4 fibre bundles expressed MFN-2 to a greater extent than all fibres isolated from the contralateral control muscle (P<0.05; Fig. 6A). While the over-expression is slightly lower in these fibres, this reflects known variation in transfection efficiency between fibres, and the random selection of fibres used for H_2_O_2_ emission and oxygen consumption determinants. Nevertheless, similar to mitochondrial respiration, over-expression of MFN-2 did not alter the rates of mitochondrial H_2_O_2_ emission in permeabilized muscle fibres (Fig. 6B). One benefit of the current approach to determine the qualitative assessment of over-expression is the potential for comprehensive correlational analysis. We have utilized this approach to examine the effect of MFN-2 on mitochondrial H_2_O_2_ emission rates, as we observed ∼5 fold variation in MFN-2 protein across all fibres. In further support of the finding that MFN-2 does not regulate mitochondrial H_2_O_2_ emission rates in mammalian skeletal muscle, across all fibres analyzed (18 control and 18 transfected) MFN-2 did not correlate with H_2_O_2_ emission rates (R^2^ = 0.02). Therefore, similar to our respiration experiments, increasing MFN-2 protein in mammalian muscle did not alter rates of mitochondrial H_2_O_2_ emission. Altogether our data suggests that over-expressing MFN-2 in healthy mammalian muscle does not affect mitochondrial bioenergetics in any capacity, however caution is warranted as we have only utilized succinate-induced reverse electron flow to asses H_2_O_2_ emission, and potentially forward electron flow and H_2_O_2_ emission from complex III may respond differently to MFN-2 overexpression.

### Perspectives and conclusions

In the current study we have assessed the direct effects of over-expressing MFN-2 in mature mammalian muscle, and find that increasing MFN-2 protein in isolation does not alter mitochondrial bioenergetics in healthy mature mammalian muscle. While this finding is at odds with the cell culture literature, there are several reports in the literature supporting the notion that altering the expression of proteins regulating mitochondrial dymanics does not affect mitochondrial bioenergetics in mature muscle. For instance, while ablating MFN-2 or MFN-1 alters the morphology of mitochondria, state III respiration in isolated mitochondria remains uneffected [22], [24]. In addition, ablation of MFN-2 in the heart does not affect proton motive force or overal performance of the heart, suggesting MFN-2 deficiency does not impact ATP production or mitochondrial bioenergetics [23]. Moreover, in the adult heart decreasing the expression of either OPA1 of MFN-2 unexpectedly increased the size of mitochondria [23], [40] while increasing the respiratory sensitivity to ADP (ie. decreased Km) without altering maximal respiration rates in permeabilized fibres [40]. Altogether with the current study, evidence is mounting to question the purported role of mitochondrial fusion proteins in regulating mitochondrial bioenergetics in mature mammalian muscle. However, the caveat to all of these studies (current study and [24], [40]) is they were designed to specifically determine the effect of altering an individual protein (MFN-2 in the current study). We have not attempted to determine the effect of over-expressing MFN-2 on mitochondrial reticulum structure, as we have not devised a method to select fibres positively transfected that remain viable for TEM or confocal imaging. Therefore, it remains possible that simultaneous alterations in fission proteins are required to observe a phenotype in mammalian muscle [18], or conversely similar to MFN-1 a chaperone protein for MFN-2 exists [42], although this remains to be determined. Therefore, future research should address these questions, as in the current study the interpretation remains focussed on the lack of a bioenergetic effect following MFN-2 over-expression. In addition, the recent report implicating MFN-2 in the regulation of endoplasmic reticulum/mitochondrial interaction [43], as well as insulin sensitivity [41], should be considered in the context of MFN-2 over-expression within muscle. It remains possible that, independent of bioenergetics, mitochondrial fusion primarily regulates the permeability transition pore opening in reponse to calcium overload in mammalian muscle [24], [40], which may have implications to apoptotic events and insulin resistance. Previous studies linking MFN-2 to mitochondrial bioenergetics have all utilized ablation approaches [7], [16], [24], suggesting MFN-2 may be required for normal mitochondrial bioenergetics. However, contrary to cell line experiments [16], [17], [18], the current data strongly suggests that increasing MFN-2 content independently does not directly influence mitochondrial bioenergetics in resting mature mammalian skeletal muscle. Therefore, it is currently unclear if the normal physiological variation in MFN-2 protein observed following training [44] and obesity [17], [37] alters mitochondrial bioenergetics.
